# Cassette deletion in multiple shRNA lentiviral vectors for HIV-1 and its impact on treatment success

**DOI:** 10.1186/1743-422X-6-184

**Published:** 2009-10-30

**Authors:** Glen J Mcintyre, Yi-Hsin Yu, Anna Tran, Angel B Jaramillo, Allison J Arndt, Michelle L Millington, Maureen P Boyd, Fiona A Elliott, Sylvie W Shen, John M Murray, Tanya L Applegate

**Affiliations:** 1 Johnson and Johnson Research Pty Ltd, Level 4 Biomedical Building, 1 Central Avenue, Australian Technology Park, Eveleigh, NSW, 1430, Australia; 2 School of Mathematics and Statistics, The University of New South Wales, Sydney, NSW, 2052, Australia; 3 The National Center in HIV Epidemiology and Clinical Research, The University of New South Wales, 376 Victoria St. Darlinghurst, NSW, 2010, Australia

## Abstract

**Background:**

Multiple short hairpin RNA (shRNA) gene therapy strategies are currently being investigated for treating viral diseases such as HIV-1. It is important to use several different shRNAs to prevent the emergence of treatment-resistant strains. However, there is evidence that repeated expression cassettes delivered via lentiviral vectors may be subject to recombination-mediated repeat deletion of 1 or more cassettes.

**Results:**

The aim of this study was to determine the frequency of deletion for 2 to 6 repeated shRNA cassettes and mathematically model the outcomes of different frequencies of deletion in gene therapy scenarios. We created 500+ clonal cell lines and found deletion frequencies ranging from 2 to 36% for most combinations. While the central positions were the most frequently deleted, there was no obvious correlation between the frequency or extent of deletion and the number of cassettes per combination. We modeled the progression of infection using combinations of 6 shRNAs with varying degrees of deletion. Our *in silico *modeling indicated that if at least half of the transduced cells retained 4 or more shRNAs, the percentage of cells harboring multiple-shRNA resistant viral strains could be suppressed to < 0.1% after 13 years. This scenario afforded a similar protection to all transduced cells containing the full complement of 6 shRNAs.

**Conclusion:**

Deletion of repeated expression cassettes within lentiviral vectors of up to 6 shRNAs can be significant. However, our modeling showed that the deletion frequencies observed here for 6× shRNA combinations was low enough that the *in vivo *suppression of replication and escape mutants will likely still be effective.

## Introduction

Human Immunodeficiency Virus type I (HIV-1) is a positive strand RNA retrovirus that causes Acquired Immunodeficiency Syndrome (AIDS) resulting in destruction of the immune system and leaving the host susceptible to life-threatening infections. RNA interference (RNAi) is a recently discovered mechanism of gene suppression that has received considerable attention for its potential use in gene therapy strategies for HIV (for review see [1-3]). RNAi can be artificially harnessed to suppress RNA targets by using small double stranded RNA (dsRNA) effectors identical in sequence to a portion of the target. Short hairpin RNA (shRNA) is one of the most suitable effectors to use for gene therapy. shRNA consists of a short single stranded RNA transcript that folds into a 'hairpin' configuration by virtue of self-complementary regions separated by a short 'loop' sequence akin to natural micro RNA (miRNA). shRNAs are commonly expressed from U6 and H1 pol III promoters principally due to their relatively well-defined transcription start and end points.

The potency of individual shRNA has been extensively demonstrated in culture and there are now several hundred identified targets and verified shRNAs for HIV [4-6]. However, it has also been shown that single shRNAs, like single antiretroviral drugs, can be overcome rapidly by viral escape mutants possessing small sequence changes that alter the structure or sequence of the targeted region [7-11]. Mathematical modeling and related studies suggest that combinations of multiple shRNAs are required to prevent the emergence of resistant strains [12-14]. There are several different methods for co-expressing multiple shRNA, including: different expression vectors [15-17], multiple expression cassettes from a single vector [5,18,19], and long single transcripts comprised of an array of multiple shRNA domains [10,20-23]. The multiple expression cassette strategy is perhaps the most useful method for immediate use due to its ease of design, assembly, and direct compatibility with pre-existing active shRNA. This strategy has been used successfully in transient expression studies with cassette combinations ranging from 2 to 7 [5,18,19,24,25].

To date, there have been limited *in silico *studies analyzing the impact of anti-HIV gene therapy [14,26]. We developed a unique stochastic model of HIV infection in CD4+ T cells to determine how many shRNAs, stably expressed in CD34+ cells, are required to control infection and the development of resistance (**manuscript in preparation**). Using our model, we simulated the development of mutations and the progression of infection for more than 13 years. Our simulations provided evidence that 4 or more shRNA can effectively suppress the spread of infection while constraining the development of resistance, which is in accord with other estimates [12-14].

Third generation and later lentiviral vector systems are currently being investigated for gene therapy applications [27-29]. These systems consist of a gene transfer plasmid, and several packaging plasmids that encode the elements necessary for virion production in the packaging cell line. The gene transfer plasmid contains a minimized self-inactivating (SIN) lentiviral carrier genome into which the therapy (e.g. multiple shRNA expression cassettes) is placed. Importantly, single pol III based shRNA expression cassettes have been incorporated into viral vectors which have been stably integrated both in culture and whole animals with effective silencing maintained over time [17,30-33]. Lentiviral vectors are now being tested in clinical trials [34,35], though they have some drawbacks described as follows.

Being derived from HIV-1, lentiviral vectors may be prone to high levels of recombination-mediated rearrangement resulting in sequence duplication or deletion [36,37]. HIV-1 reverse transcriptase (RT) is especially suited to 'jumping' between duplicated regions, since it requires a similar functionality to copy the LTRs [38-40]. It is thought that repeat deletion mostly occurs during retroviral minus strand synthesis when the growing point of the nascent minus strand DNA dissociates from the first RNA template (template switch donor) and re-associates to a homologous repeat in the same or a second template (template switch acceptor) [36,41]. Intermolecular template switching amongst the 2 genomes co-packaged in each viral particle occurs between ~3 - 30 times for every infection [36,42,43], making it more common than base substitutions (occurring at ~3 × 10^-5 ^mutations per base per infection [44]). This implies that every HIV-1 DNA is recombinant, though recombination will only produce a change if a cell is multiply infected, which is rarer. Previous studies of different double repeats have shown a correlation between the length of the repeated sequence and the frequency of deletion [37]. However, the association between the number of repeated units > 3 and deletion frequencies has not yet been studied. ter Brake *et. al*. have recently shown that one or more repeated shRNA expression cassettes in lentiviral vectors may be deleted during the transduction process [45]. They independently transduced 11 double shRNA combinations and 37 triple shRNA combinations and found that 77% were subject to deletion. Though a small scale study, their findings pose a potentially major problem to using multiple shRNAs for gene therapy in a repeated cassette format. It follows that the deletion of 1 or more shRNAs from multiple shRNA therapies may decrease protection and increase the likelihood for development of resistant viral strains.

The primary aim of this study was to characterize on a larger scale the frequency of deletion and its relationship to the number of cassettes combined for combination lengths of 2 to 6 shRNA expression cassettes. We also aimed to mathematically model the outcomes of different frequencies of deletion in gene therapy scenarios. We found that all combinations were subject to deletion, but found no correlation between the extent of deletion and combination length. Our models of semi-deleted combinations of 6 shRNAs indicate that combinations more extensively deleted than observed here (for 6× shRNAs) may still suppress viral replication and the emergence of shRNA-resistant strains.

## Results

### Selecting combinations of up to 6

We have previously analyzed over 8000 unique 19 nucleotide (nt.) HIV-1 targets, and calculated their level of conservation amongst almost 38000 HIV gene sequence fragments containing 24.8 million 19 mers [6]. Using our conservation 'profile' method, we characterized 96 highly conserved shRNAs using fluorescent reporter and HIV-1 expression assays. Ten of these (shRNAs #0 - 9) were selected for assembly into 26 multiple shRNA combinations from 2 to 7 shRNAs using a repeated expression cassette strategy with multiple H1 promoters (**manuscript submitted**). We selected one 6× shRNA combination along with its series of related intermediate combinations and corresponding single shRNA vectors to test herein. This comprised shRNAs #3 (Pol 248-20), #8 (Vpu 143-20), #9 (Env 1428-21), #2 (Gag 533-20), #7 (Tat (x1) 140-21), #6 (Vif 9-21) (Table 1), and the following combinations: 2.2 (shRNA #3.8) {*the combination name representing a 2 shRNA combination (2.×), and the second variant made in the original study (x.2), followed by its component shRNAs separated by periods*}, 3.2 (#3.8.9), 4.3 (#3.8.9.2), 5.3 (#3.8.9.2.7) and 6.3 (#3.8.9.2.7.6). We were most interested in combinations of 6 shRNAs as we have previously shown that with this number of shRNAs we can assemble a therapy with at least 4 shRNAs matched to all known clade B variants (**manuscript submitted**).

**Table 1:** The 6 shRNAs

**#**	**Target**	**p-2,1**	**Core 19 mer (p0)**	p+1,2 *	**Loop**	**T.sp.**
**2**	Gag 533-20	AG	GAGCCACCCCACAAGATTT	A**A**	TCTCGAGT	
**3**	Pol 248-20	AG	GAGCAGATGATACAGTATT	A**G**	CCTCGAGC	
**6**	Vif 9-21	AA	CAGATGGCAGGTGATGATT	GT	ACTCGAGA	
**7**	Tat (x1) 140-21	CT	ATGGCAGGAAGAAGCGGAG	AC	ACTCGAGA	A
**8**	Vpu 143-20	AA	GAGCAGAAGACAGTGGCAA	T**G**	CCTCGAGC	
**9**	Env 1428-21	AA	TTGGAGAAGTGAATTATAT	AA	ACTCGAGA	

The 6 shRNAs came from our previous study of 96 highly conserved shRNAs for HIV-1. The shRNAs had either 20 or 21 bp stems (as indicated in the shRNA name) built around a 19 bp p0 core placed at the base terminus of the shRNA. Nineteen bp targets were selected using a conservation profile method, where the 2 bases immediately upstream (p-2,1) and downstream (p+1,2) of the 19 bp target were also taken into consideration when estimating conservations. The identity of the sequence external to the shRNA stem was adjusted, where possible, to correspond to the flanking sequence in the target. Each shRNA consisted of a stem made from the 19 mer p0 core (shown) plus the p+1 nucleotide for 20 bp stems, or both p+1, 2 nucleotides for 21 bp stems, connected by the indicated loop. shRNAs for which the last base of the anti-sense stem was 'T' also included a 'termination spacer' (T.sp.) so as to prevent premature termination via an early run of 'T's. This nucleotide was always the complement of the first nucleotide of the p-1 position (but never a 'T'), so that if included in the processed siRNA product(s) it was also matched to the target. * The bases shown in bold (the p+2 position) were not a part of the stem for these shRNAs as they only had 20 bp stems. The shRNAs with 21 bp stems included both p+1, 2 positions.

### Repeated sequence in our multiple shRNA expression cassette configuration

**Figure 1:**
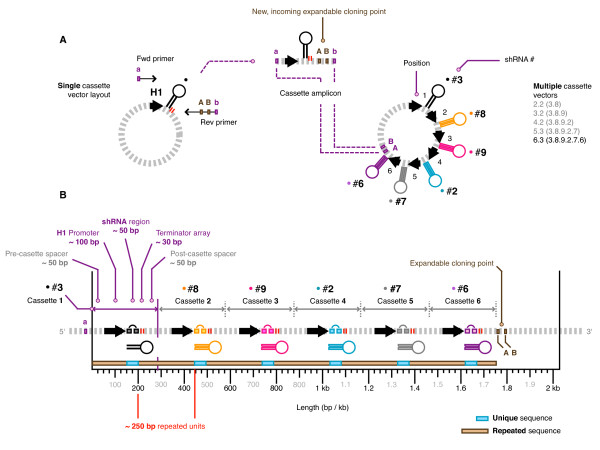
**shRNA cassette configuration**. (**A**) Each single shRNA was originally expressed from a human H1 (pol III) promoter in separate vectors. Multiple cassette combinations were made by PCR amplifying each promoter-shRNA-terminator (plus ~100 bp of common flanking sequence) as a self-contained expression cassette, and sequentially inserting them into a single vector via an infinitely expandable cloning strategy. The PCR amplified shRNA expression cassette was digested with 'a' (*Mlu I*) and 'b' (*Asi SI*) restriction enzymes (REs) and was ligated to the recipient vector opened up with 'A' (*Asc I*) and 'B' (*Pac I*) REs destroying the original 'a', 'A', b', and 'B' sites in the process. The newly created vector has the 'A' and 'B' sites reconstituted via the incoming donor fragment, ready for insertion of subsequent cassettes. The series selected for this study begins with shRNA #3, followed by #8 to make combination 2.2 (shRNA #3.8). Additional shRNAs were added in order to make the combinations 3.2 (#3.8.9), 4.3 (#3.8.9.2), 5.3 (#3.8.9.2.7) and 6.3 (#3.8.9.2.7.6). (**B**) The average cassette length was ~300 bp long, of which 250 bp (83%) was repeated since each expression cassette was transferred into combination using generic primers.

Our combination vectors were constructed in lentiviral vectors using a novel cloning strategy that theoretically enables an infinite number of cassettes to be sequentially inserted [46]. Each expression cassette was transferred from identical single shRNA expression vectors (barring the unique shRNA, of course) into combination vectors via PCR with generic primers (Figure 1a). This made assembly swift, but also resulted in a large amount of sequence repeated in each cassette. The average cassette length was ~300 bp long, of which 250 bp (83%) was repeated (Figure 1b). This does not consider the identical short 8 bp loop encoding sequence for each shRNA (< 3%) due to its small size and relative placement. The only unique sequence per cassette with this design was contributed by the sense and anti-sense stems of each unique shRNA.

### Challenging stably infected single shRNA populations with HIV-1

**Figure 2:**
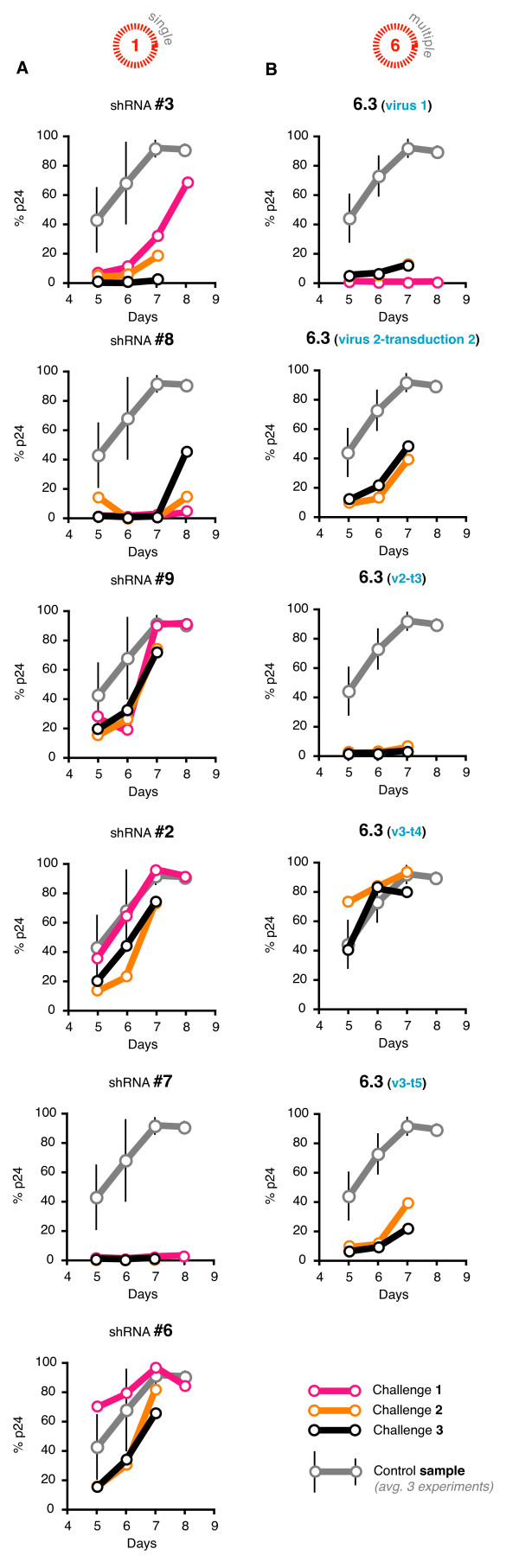
**Inconsistent challenge results from repeated stable transductions of 6.3**. (**A**) We challenged G418 selected CEMT4 polyclonal populations of each of our 6 single shRNA vectors with HIV-1. Suppressive activities were inferred by intracellular p24 levels measured between 5 and 8 days later. Each population was assayed in 3 independently repeated experiments. A control vector expressing a single shRNA unmatched to HIV-1 was also tested 3 times (grey points), with the average values of 3 experiments and 95% confidence intervals (CI) shown. (**B**) Five separate 6.3 polyclonal populations were generated through independent **t**ransductions (t1 to t5) using 3 different lenti**v**iral batches (v1, 2, and 3). Each population was similarly selected and challenged in 3 independently repeated experiments with HIV-1. The control vector was a combination of 6 shRNAs unmatched to HIV-1 that were assembled in the same format as 6.3 (grey points), with the average values of 3 experiments and 95% confidence intervals (CI) shown.

We infected CEMT4 cells with virions made from each of our 6 single shRNA lentiviral gene transfer plasmids to create 6 different stably integrated polyclonal populations each containing a single shRNA. The suppressive activity of each population was measured with an HIV-1 challenge assay. In this assay, the target populations were infected with the NL4-3 strain at an MOI of 0.0004, and the amount of viral replication was inferred by intracellular p24 levels measured between 5 and 8 days later. Suppressive activities were calculated by comparing the p24 levels of the shRNA containing populations to the p24 levels from untransduced CEMT4 cells (Figure 2a). Some of our selected shRNA populations exhibited little or no activity when comparing the p24 levels to a population stably infected with a non-specific shRNA (a backwards control sequence unmatched to HIV-1). For others, the suppressive effect was overcome at days 7 - 8 due to excessive HIV replication killing all infected cells and saturating our capacity to measure p24. However, shRNAs #3, 7 (in particular) and 8 showed strong activity that was maintained for the course of the assay.

### Challenging stably infected 6× shRNA populations with HIV-1

We similarly created a stably integrated polyclonal population for our chosen combination of 6 shRNAs (6.3: 3.8.9.2.7.6). Our first challenge result was encouraging, with strong suppression of viral replication over all time points measured (Figure 2b). However, repeated tests using up to 3 different virus batches and 5 different stably integrated polyclonal populations showed variable results. Repeated challenges of these populations showed different levels of activity, ranging from inactive to extremely active. These findings may fit with a recently published report that one or more cassettes may be deleted during transduction, resulting in alterations in observed suppressive activities [45]. Importantly, this work shows that multiple cassette combinations like ours cannot be reliably analyzed via polyclonal populations.

### Up to 100 clonal populations for each 2 - 6 shRNA combination

To investigate the extent of deletion we created several sets of individually transduced clonal cell lines. These sets included our combination of 6 shRNAs (6.3), and its corresponding sub-combinations of 2 to 5 (2.2, 3.2, 4.3, and 5.3) so we could assess the relationship between cassette deletion and combination length. We performed pooled transductions for each combination and serially diluted them into more than 100 single cell populations per combination which we expanded under G418 selection. We were able to recover 100 expanded populations for 2.2, 5.3 and 6.3, but only 83 populations for 3.2, and 48 for 4.3. Approximately 10 - 12 weeks after transduction the populations were selected and sufficiently expanded to be harvested for their DNA.

### Testing our clonal populations for deletion via PCR and dot blot arrays

**Figure 3:**
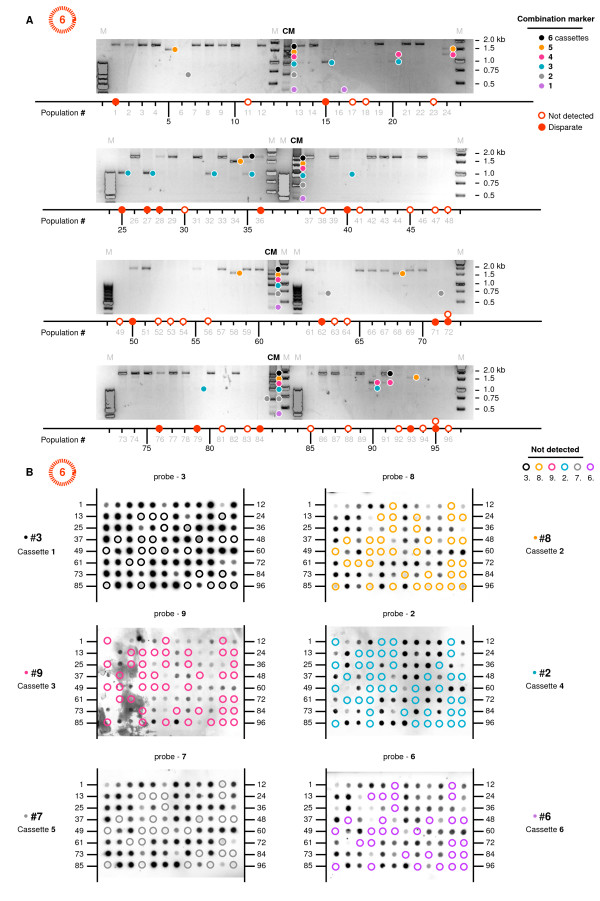
**PCR and dot blot methods to assay combination lengths and composition**. All samples were amplified across the multiple cassette region via PCR and the products were separated with gel electrophoresis. All samples were also subject to a control G418 resistance gene (neo^r^) amplification reaction to verify the integrity of the extracted sample (data not shown; all samples positive). The PCR products were immobilized onto membranes and probed using shRNA-specific probes to characterize the component shRNAs of each amplified product. This figure shows a representative example of (**A**) the raw PCR separations and (**B**) dot blot exposures for the first 96 6.3 populations amplified and probed for shRNAs #3 and #8. *n.b. smaller products were poorly amplified with the reaction conditions optimized for longer products, making visualization sometimes difficult*. Several samples had multiple bands (#20 - 4,3; #24-5,4; #35-6, 3; #90-4, 3; #91-6, 4), for most of which the larger size was more readily detected. These were scored as the largest size. CM: **C**assette **M**arker (a custom 1-6 cassette marker made by PCR of the plasmid stocks). M: size **M**arker (standard 100 bp and 1 kb DNA ladders, Invitrogen). Dot blots were scored qualitatively as detected (+ve)/not detected (-ve) above background levels, taking into account the presence/absence of PCR products detected by gel electrophoresis for weakly detected bands. Probe #9 bound the least efficiently; some weakly detected products seen on the original films may not be apparent in the reproduced images. Samples with disparate results between the two methods correlated with poorly amplified products that were difficult to visualize with electrophoresis and were consequently weakly detected by dot blot analysis (red dots).

**Figure 4:**
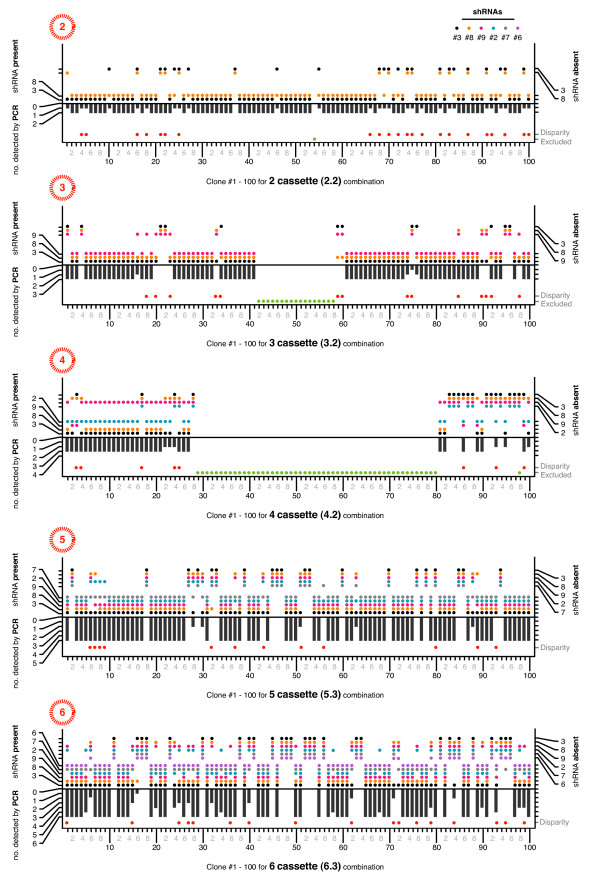
**500 stable transductions of 2.2, 3.2, 4.3, 5.3 and 6.3**. The results from both PCR and dot blot assays were summarized into 3 panel plots for each set of populations, with individual cassettes shown as dots in the top two panels (not detected and detected cassettes respectively), and the combination length measured by electrophoresis in the bottom panel. Some samples, mostly for 3.2 and 4.3, were excluded from analysis because there were either no colonies recovered from selection, or the neo^r ^control PCR was negative (green dots). Samples with disparate results between the two methods are indicated by red dots. The data shown is representative of 2 independently repeated amplification and detection experiments.

All samples were amplified across the multiple cassette region via PCR using standard Taq reactions for combinations of 2 shRNAs, and a specially adapted Pfu reaction for combinations > 2 [46]. By separating the PCR products with gel electrophoresis we were able to discriminate between all combination sizes of 0 to 6 shRNAs. All samples were also subject to a control G418 resistance gene (neo^r^) amplification reaction to verify the integrity of the extracted sample. All but 3 samples were positive for neo^r^. The PCR products were also immobilized into arrays of 100 dots onto as many membranes as there were shRNAs in each combination, and probed using shRNA-specific probes (Figure 3). This dot blot technique enabled us to characterize the component shRNAs of each amplified product. The results from both assays were summarized into 3 panels for each set of populations, with individual cassettes shown as dots in the top two panels (not detected and detected cassettes respectively), and the combination length measured by electrophoresis in the bottom panel (Figure 4).

**Figure 5:**
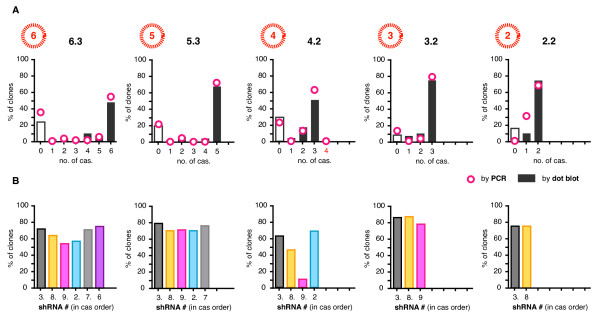
**The no. of cassettes lost and the frequencies of shRNAs detected**. (**A**) The total number of cassettes detected (e.g. 1-6 for 6.3 populations) were tallied for each clonal population across each combination set (i.e. 2.2, 3.2, 4.3, 5.3 and 6.3) and expressed as a percentage of the total number of populations within each set (e.g. 100 clonal populations analyzed for 6.3). Tallies for both PCR (bars) and dot blots (circles) shown. (**B**) The individual cassettes detected by dot blot were tallied as percentages of the populations, and shown in order in which the cassettes are arranged in each combination.

All combination lengths were subject to deletion, with 28 - 36% of 6.3 populations, 6 - 17% of 5.3, all 4.3 populations, 6 - 18% of 3.2, and 12 - 18% of 2.2 populations having one or more entire cassettes deleted. The ranges denote the slightly differing estimates from both methods of analysis and discounted samples with no products detected from either method (which ranged from 2 - 26%). If our figures were increased by the number of undetected samples being tallied as having 1 or more deletions then the maximum deletion frequency observed here would be 52% for 6.3. Three and 5 shRNA combinations were the least affected (6 - 12%), whereas 100% of 4 shRNA populations showed some deletion. On average 16% of samples had disparate results between the 2 methods. These correlated with poorly amplified products that were difficult to visualize with electrophoresis and were consequently weakly detected by dot blot analysis. This was not unexpected, as amplifying repeated shRNA expression cassettes by PCR is technically challenging, even though we used a PCR method specifically developed for repeat sequences [44]. The number of cassettes deleted was spread across all possible sizes (e.g. deletions across the 6.3 populations ranged from 1 to 5 cassettes), with the exception of 4 shRNA populations which mostly had shRNA #9 in the third position deleted leaving 3 remaining cassettes (Figure 5a). The 4, 5 and 6 shRNA populations had greater deletions from their central positions (Figure 5b). Barring one disparate sample, there were no populations of > 2 shRNAs that had both terminal cassettes simultaneously deleted.

### Setting modeling parameters

We modified our previous *in silico *model of HIV-1 infection in the presence of multiple shRNAs to test the hypothesis that loosing one or more shRNAs may affect treatment success. Our model simulated infection over 13 years for 343000 cells contained in a 3-dimensional space that represented lymphoid tissue where the influence of cell proximity on viral transmission was considered. We set the number of CD34+ progenitor cells transduced ('marked') at 20%. Mutated viruses had fitness reduced to 99% (c.f wildtype at 100%). Individual shRNAs were modeled as being 80% effective, with multiple shRNAs assumed to provide an independent effect of 100 × (1 - (1 - 0.8)^n^) %, where 'n' was the number of shRNAs present per combination or semi-deleted shRNA profile. We included calculations to ensure that all cells killed by infection were replaced by cells from one of two sources. This enabled us to follow the progression of infection for 13 years without the model crashing due to loss of cells. The sources for replacement cells were either (1) cells newly maturing from the thymus or (2) from division of neighbouring CD4+ cells that either contained shRNAs (i.e. originated from the original transduced CD34+ population), or were unmodified (i.e. without shRNAs). If replacement cells were derived from neighbouring cells, they retained the same shRNA profile of the parental cell if it was descended from a transduced cell, or had no shRNAs if the parent cell came from an unmodified lineage. However, if the replacement cells maturated from the thymus, then the shRNA profile was randomly assigned in accordance with the range of semi-deleted shRNA combinations being evaluated per scenario (as described above). All scenarios were initiated with a single wildtype virus sequence, and were pre-run for 100 days to mimic the natural course of infection prior to treatment with gene therapy. This enabled HIV to disseminate, accumulate mutations and develop into a pool of variant strains to simulate natural HIV diversity. Transduced cells were introduced into the model after HIV diversity was established. Only mutations occurring within shRNA target sites that would confer resistance to the shRNA were tracked. See our **Methods **for additional detail.

### Modeling the impact of cassette deletion on the progression of infection

We simulated 7 scenarios containing 6 or fewer shRNAs. Scenarios 1, 2, and 3 modeled control combinations of 6, 4, and 2 shRNAs respectively, in which no cassettes were deleted. Scenarios 4 - 7 each modeled different amounts of deletion for combinations of 6 shRNAs. In scenario 4, 90% of transduced cells contained an intact combination of 6 shRNAs, and the remaining 10% of cells were evenly distributed with 5 - 1 cassettes being deleted, summarized as: **s4**: 6 (90%), 5 - 1 (2% each). The other scenarios were **s5**: 6 (50%), 5 - 1 (10% each); **s6**: 6 - 5 (0% each), 4 (90%), 3 (3%), 2 - 1 (2% each); and **s7**: 6 - 5 (0% each), 4 (50%), 3 (20%), 2 - 1 (15% each) (Table 2). The positions of the deleted cassettes were randomly assigned (i.e. 1 - 6), since deletions distributed across all possible positions maintained an even diversity of targets in the entire population of transduced cells. For example, there are 15 different combinations of 4 shRNAs (shRNA profiles) possible when deleting any 2 shRNAs from a fixed combination of 6 shRNAs (as determined by the combinatorial choose function: n!/(k!(n - k)!); in this case 6!/(4!(6-4)!)), of which any one was randomly assigned. This closely approximated our practical observations of deletions which were spread across all positions, excluding ~5% of all possible profiles in our modeling which had both terminal positions simultaneously deleted (which we did not observe experimentally).

**Table 2:** shRNA profiles for each scenario modeled

	**% of cells with combinations of the indicated shRNA number per scenario**					
**Scenario**	**6×**	**5×**	**4×**	**3×**	**2×**	**1×**
**1**	100	0	0	0	0	0
**2**	-	-	100	0	0	0
**3**	-	-	-	-	100	0
**4**	90	2	2	2	2	2
**5**	50	10	10	10	10	10
**6**	0	0	90	4	3	3
**7**	0	0	50	20	15	15

The proportion of cells containing each shRNA profile within the marked population (which constitutes 20% of the total number of cells in the model).

**Figure 6:**
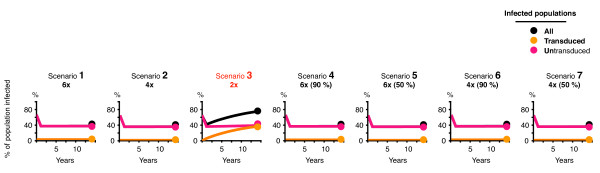
**Modeling different degrees of repeat deletion for combinations of 6 shRNAs**. (**A**) The 7 scenarios modeled, showing the progression of infection via the % of transduced cells, untransduced cells, and all cells (transduced + untransduced) that were infected over the course of the simulation (~13 years).

We first modeled a control scenario of untreated cells (i.e. no gene therapy) exposed to HIV, however, the simulation ended prematurely at ~500 days when 100% of cells were infected. The best-case treatment scenario in which 100% of transduced cells contained an intact combination of 6 shRNAs (s1) offered only marginally better protection than the worst-case semi-deleted scenario in which 50% of cells had 4 shRNAs or fewer (s7) (Table 3). In this comparison the number of infected cells increased from 35 to 40% of the total monitored after 5000 days of simulation. Surprisingly, the total number of uninfected cells remained similar across all scenarios with 4 or more shRNAs (Figure 6). In these cases, more than 98% of the uninfected cells were from the transduced population, indicating that even with extensive deletions a high level of protection was maintained. The small increase in the number of infected cells that correlated with increasing deletions was mostly from wildtype infections in transduced cells unable to suppress replication (i.e. to few shRNAs). For example, there was a 43 fold increase in wildtype virus infections (0.1 to 4.3%) between the most extreme scenarios (s1 vs. s7). There was also a 20 fold increase in the small proportion of transduced cells that were infected with a mutated virus that was resistant to a single shRNA (0.005 - 0.01%). In contrast, the combination of 2 shRNAs alone - even without any deletions - was ineffective in suppressing replication, with ~75% of the entire population infected after 13 years. Interestingly we observed no strains that developed resistance to more than 2 shRNAs either sequentially or simultaneously in any scenario.

**Table 3:** Final proportions of cells (% of the total) after 13 years

	**Uninfected cells**		**Infected cells**			
	**Untransduced**	**Transduced**	**Untransduced**	**Transduced**		
**Scenario**				**m = 0**	**m = 1**	**m = 2**
1	1.3	63.5	35.1	0.1	0.005	0.0003
2	0.7	61.8	35.2	2.4	0.08	0
3	0.1	25.7	38.2	35.6	0.5	0
4	1.2	63.5	35.0	0.3	0.01	0
5	0.9	62.6	35.1	1.4	0.04	0.0003
6	0.6	61.5	35.1	2.7	0.08	0
7	0.4	59.8	35.4	4.3	0.1	0.001

The percentage of untransduced and transduced cells that are uninfected and infected after 5000 days (13 years) of modeling. The resistance profiles for the transduced cells that are infected are also shown, divided into the percentage of total cells infected with wildtype virus (m = 0) and virus containing mutations that confer complete resistance to either 1 (m = 1) or 2 shRNAs (m = 2). n.b. we observed no strains that developed resistance to more than 2 shRNAs.

## Discussion

### Our results in context

We observed deletion frequencies of 2 - 36% for 2, 3, 5 and 6 cassette combinations with ~250 bp of repeated sequence per cassette, and ~50 bp of unique sequence separating each repeat. While the central cassette positions were the most frequently deleted there was no progressive correlation between the frequency or extent of deletion and combination length, though combinations of 6 were the most affected. In contrast, all samples from our 4 cassette populations had one or more deletions. Why this set showed significantly more deletions than any other is unclear to us. Interestingly, the 4 cassette populations also had the lowest recovery rate following transduction with less than half surviving selection. We know of no reason why our combination of 4 should be more susceptible to repeat deletion compared with other combinations. This result may be due to an experimental anomaly or a deleterious response characteristic of this particular combination. Others have reported deletion frequencies of 77% for 2 and 3 shRNA cassette combinations with repeated units of comparable size and spacing to ours [45], and 7%, 20% and 87% for double combinations with adjacent non-shRNA repeated units 117, 284 and 971 bp long [37]. Our frequencies were on average between 56 - 62% lower than that reported by ter Brake *et. al*. [45], but were in a similar range for the corresponding cassette size to that reported by An and Telesnitsky [37].

### Fitting our observations to the mechanism of rearrangement

Our observation that no populations of > 2 shRNAs had both terminal cassettes simultaneously deleted while central cassettes remained intact is in accord with ter Brake *et. al*. [45], and consistent with the proposed mechanism of repeat deletion. Assuming that repeat deletion occurs via RT transcribing part of one genome and swapping to a homologous region of second genome for completion [36,42], then all rearranged constructs must retain at least the first or the last cassette. Our suppressive activity tests via HIV-1 challenge assays also support the notion that rearrangement occurs after viral production, since identical viral preparations yielded different results from repeated transductions.

### Are shRNA cassettes more prone to recombination than non-structured templates?

Previous work has shown that sequences with strong secondary structures may induce more mutation and recombination in HIV and other retroviruses than homologous sequences alone [47,48]. It is thought that strong secondary structures can cause the RT to pause and or slow the rate of polymerization, both of which are known to increase the incidence of template switching [36]. Whether this applies specifically to shRNA expression cassettes is not known. We have previously generated a small scale set of 22 clonal populations transduced with a 6 cassette combination comprised of empty expression cassettes (i.e. repeated H1 promoters without shRNAs), and saw one or more deletions in 9 of these samples (41%) (**data not shown**). This suggests that deletion in the context of our vector design is independent of the presence of shRNA sequences, which again is in accord with the underlying mechanism of deletion. This requires validation though, as our control analysis was too small to draw conclusions of relative deletion frequencies between templates with and without shRNA expression cassettes.

### The impact of the space between repeated units

Interestingly, it has been shown that deletion rates in murine leukemia virus (MLV) increase when repeat regions are separated by a spacer [49]. Why this would facilitate template switching is unclear to us. Our design incorporated ~100 bp of spacer sequence between transcriptional units, though this formed a part of each ~250 bp repeated unit. We included this extra sequence in the event that the space between cassettes may reduce interference between multiple transcription complexes attempting to transcribe shRNAs from adjacent cassettes, though this assumption remains untested. There is a lot of scope to further study the relationship between the length of inter-cassette spacers and deletion frequencies.

### Reducing similarities in repeated sequences

Previous work suggests that retroviral recombination may be more permissive of mismatched repeats than either bacterial or mammalian recombination. In one study of double 156 bp repeats (separated by ~1.5 kb), incremental and evenly distributed differences ranging from 5 to 42% were added into one copy without changing the second [50]. As little as 5% difference between repeats decreased deletion frequency by 65% cf. identical repeats, an 18% difference reduced deletion frequency to 5%, and a 27% difference eliminated deletion events. However, in other systems where differences were not evenly distributed, as few as 12 repeated nucleotides may be sufficient for homologous recombination to occur, albeit at low frequencies [42,51,52]. By comparison, a 16 - 19% mismatch between sequences in bacteria and mammalian cells can reduce intra-chromosomal recombination by 100 to 1000 fold (cf. the 20 fold change at 18% mismatch for retroviruses) [50,53,54]. None-the-less, these studies suggest that it may be possible to use 'near-identical' repeated cassettes to reduce recombination-mediated deletion if strategic sequence changes could be introduced without interfering with their function.

### Methods to 'get around' rearrangement

The most obvious solution to overcome recombination-mediated deletion is to eliminate repeated sequences. Others have shown the usefulness of such an approach with 4 shRNA expression cassettes by replacing repeated H1 promoters with a medley of promoters; H1, mH1 (mutated), U6, mU6 (murine), 7sk and U1 (n.b. pol II) [24,45]. Their improved constructs performed more reliably under repeated transduction conditions than the equivalent all H1 constructs. Although the most straightforward approach, it is presently limited by the small number of promoters suitable for shRNA expression and stacking in lentiviral vectors (e.g. compact promoters such as the H1, U6 and 7sk pol III promoters). However, it is likely that other suitable promoters remain to be discovered. It may also be possible to develop new variations of the current promoters through strategically introduced point mutations, or to use orthologous promoters that are sufficiently different [24]. As few as 5 single base changes in the H1 promoter would equal a 5% difference, and potentially a 65% reduction in recombination-mediated deletion [50]. More ambitious solutions would be engineering or screening for a replacement RT with impaired strand exchange capabilities - though this may negatively impact on the LTR duplication/exchange events required for vector integration.

### The outcomes of our modeling

Overall, our modeling suggests that cells transduced with a combination of 6 shRNAs need only retain 4 or more shRNAs in at least 50% of cells to offer similar protection to an undeleted combination of 6. This is sufficient to effectively suppress the development of cells that contain multiple-shRNA resistant virus to < 0.1% of the total population after 13 years (343000 cells monitored). Interestingly, this is estimated to be even lower than the number of cells expected to harbor multiple-shRNA resistant virus that would exist in a similar sized population of entirely untreated cells (i.e. unexposed to selective pressure) (< 0.1% cf. < 1%) (**manuscript in preparation**). Our findings extend the conclusions within the original model, which indicated that at least 4 shRNAs in 100% of cells could suppress the development of resistance to < 0.1%. Provided sufficient numbers of CD4+ T cells are regenerated from the thymus so that the population of modified cells in the periphery is not limited to just a few combinations of 4 shRNAs, then the randomness of deletion serves to duplicate the situation where all cells contain the full complement of 6 shRNAs. Emerging strains resistant to any one particular sub-combination are likely to be suppressed by the other sub-combinations of different identity expressed in other cells. In practical terms, our model indicates that even a significant loss of shRNAs in a portion of transduced cells will not significantly decrease the efficacy of treatment nor allow resistant viral strains to emerge (assuming randomness as indicated).

### The limitations of our model

The outcomes of our model may by limited by some of the underlying assumptions. We set individual shRNA efficacy conservatively at 80%. Though shRNAs #3, #7 and #8 were suitably active (> 80%), our challenge results here showed that shRNAs #9, #2, and #6 were likely less than 80% active against replicating virus. Interestingly our previous reporter-based assays indicated that all 6 shRNAs were suitably active (**manuscript submitted**). It will be important to incorporate only the most active shRNAs in future combinations. In our model we also considered the suppressive effects of more than 1 shRNA to be multiplicative. While there are reports multiple shRNAs can have a higher combined suppressive activity than the corresponding single shRNAs [5,24,55], this is likely to be dependent on expression at sub-saturating levels which consequently may also lessen the individual suppressive activities of the component shRNAs. Thus, *in vivo *combined suppressive activity may not be as strong as modeled here. Finally, we only tracked mutations that occurred within the shRNA target sites. However, base changes adjacent to the target site can lead to structural alterations in the target site which confer resistance, which means we may have discounted some mutations that could have potentially lead to resistance [9]. Altering any of the above parameters in our model will likely affect the outcomes predicted by our simulations.

## Conclusion

Even though we observed significant deletions for combinations of all sizes, the deletion frequency for combinations of 6 shRNAs was well within the range predicted by our modeling to still confer effective suppression of viral replication and prevent the emergence of viral escape mutants. Overall, our results support the conclusion that resistance to gene therapy is unlikely to develop when adequate protection is provided. However, it is likely that designs prone to recombination-mediated deletion would be justifiably questioned by gene therapy regulatory bodies (e.g. the FDA), due to unpredictable variability in the product introduced into patients. Given the choice, alternative designs that minimize the amount of sequence repeated in adjacent expression cassettes would be better suited to future constructions.

## Methods

### Target sequences and multiple cassette expression vectors

Briefly, we analyzed over 8000 unique 19 nucleotide (nt.) HIV-1 targets, and calculated their level of conservation amongst almost 38000 HIV gene sequence fragments containing 24.8 million 19 mers. We selected 96 highly conserved targets and made shRNAs of 20 and 21 bp stems using a Phi-29 primer extension method [56], which we then characterized using fluorescent reporter and HIV-1 expression assays. Ten of these (shRNAs #0 - 9) were selected for assembly into 26 multiple shRNA combinations from 2 to 7 shRNAs. Combinations were assembled in a repeated expression cassette format with multiple H1 promoters using an infinitely expandable cloning strategy for construction [46]. The full details of the selection of shRNA target sequences and the assembly of multiple shRNA combination vectors has been described elsewhere [6] (and **manuscripts submitted**).

### Lentiviral (virion) production

Gene therapy virions were produced in 293AAV cells (Cell Genesys) via calcium phosphate transfection (Clontech) of the 4 lentiviral component plasmids: the shRNA containing transfer plasmid and the 3 packaging plasmids pKgagpol (Gag-Pol), pKrev (Rev) and pK.G (VSVG envelope) at a mass ratio of 20 (30 μg): 13 (19.5 μg): 5 (7.5 μg): 7 (10.5 μg) respectively. The cells being transfected were seeded at 15 × 10^6 ^in a T225 cm^2 ^flask (Corning) 24 hrs. prior to transfection. The transfection media (DMEM (Invitrogen) containing 10% FBS (Fetal Bovine Serum) and chloroquine (Sigma)) was replaced with serum free media VP-SFM (Invitrogen) 12 - 24 hrs. post-transfection and VCM (Virion Containing Medium) was harvested/concentrated 24 hrs. later by centrifugation and filtration through 0.2 μm filters.

### Lentiviral transduction, colony expansion and harvesting

Non-tissue culture treated 6 well plates were coated with Retronectin™ (Takara Bio Inc.) at 25 μg/ml in 2 ml/well and kept at 4°C for 24 hrs. (prior to transduction). Transductions were performed by first blocking the coated plates with 2% BSA (Bovine Serum Albumin) PBS (Phosphate-Buffered Saline; Invitrogen) for 30 min., followed by application of neat VCM at 2 ml/well and centrifuged at 2000 rpm (32°C) for 1 hr. The first-loaded VCM was replaced with fresh VCM together with CEMT4 cells (NIH AIDS Research and Reference Reagent Program) at 5 × 10^5 ^cells/ml in 2 ml/well, i.e. 1 × 10^6 ^cells/transduction/well, and the plates were centrifuged at 2000 rpm (32°C) for 1 hr. prior to incubation at 37°C. After 48 hrs. cells were put under selection with G418 at 800 μg/ml (Geneticin, Gibco), and kept under selection for 4 weeks and expanded into T25 cm^2 ^and T75 cm^2 ^flasks (Corning) as necessary. Once selected, the pooled populations for each 2 to 6 cassette combination were cloned out into 10× 96 well plates per combination by limiting dilution at an estimated 0.5 cells per well. In practice, many wells were empty and few wells contained more than 1 cell (typically less than 10%). On average, 10 to 50% of wells yielded suitable single colonies. Two weeks later 100 suitable clonal populations for each combination (*n.b. less than 100 populations were recovered for combinations of 3 and 4*) were moved out into 24 well plates and progressively expanded into individual T25 cm^2 ^and T75 cm^2 ^flasks as required. Each sample population was harvested into several replicate pellets from T75 cm^2 ^sized cultures. We found that the quality of our sample preparations was critically important for the subsequent success of PCR analysis. Samples were harvested using the DNAeasy kit (Qiagen) according to the manufacture's instructions, except we used a lower amount of starting material (to avoid sample contamination through overloaded columns), incorporated additional pellet washing steps prior to column loading (to minimize serum contamination), and eluted the extracted DNA samples with 2× 100 μl elutions of H_2_O to a final extraction volume of 200 μl (to maximize yield and dilute impurities).

### Preparation of HIV stocks

HIV stocks for infection were prepared by seeding low passage no. HEK293a cells (sourced from the American Type Culture Collection) [ATCC: **CRL-1573**] at 10 × 10^6 ^cells in a T225 cm^2 ^flask and transfecting the following day with 30 μg HIV-1_NL4.3 _(NIH AIDS Research and Reference Reagent Program) using Lipofectamine 2000™ (Invitrogen) at a DNA: Lipofectamine 2000™ ratio of 1: 2.5, following the manufacturer's protocol. VCM was harvested 2 days later and spun for 10 min. at 400 g to clear cells. 1 ml of VCM was used to prepare CEMT4-adapted HIV (HIV derived from CEMT4s and thus better suited to infecting CEMT4s in subsequent experiments) by infecting 1 × 10^6 ^CEMT4 cells and harvesting VCM 8 days later by centrifugation for 10 min. at 400 g to clear cells. VCM titer was determined by infecting 1 × 10^6 ^pelleted (200 g for 5 min.) CEMT4 cells with 10 fold serial dilutions of VCM, and incubating at 37°C for 2 hrs. with intermittent agitation every 30 min. Unattached virus was removed by washing in 10 ml of RPMI (Invitrogen) +10% FBS and centrifuging at 200 g for 5 min. Pelleted HIV-infected cells were resuspended in 10 ml of RPMI and 2 ml was transferred to 5 wells of a 24 well plate (Corning). Cultures were further incubated at 37°C in 5% CO_2 _and scored for syncytia formation between days 8 - 11. Viral titer was calculated using the Reed-Muench method for estimating 50% endpoints [57].

### HIV-1 challenge assay

The CEMT4 cell lines with stably integrated shRNA vectors were seeded at 3 × 10^5 ^cells/ml 2 days prior to HIV infection so that cells were growing logarithmically and were > 85% viable on the day of HIV infection. 1 × 10^6 ^cells were pelleted (200 g for 5 min.) and resuspended in 1 ml of HIV-1_NL4.3 _VCM (of the appropriate dilution, see above) at an estimated MOI of 0.0004 and incubated at 37°C for 2 hrs. with intermittent agitation every 30 min. Unattached virus was removed by washing in 10 ml. of RPMI (Invitrogen) with 10% FBS followed by centrifugation at 200 g for 5 min. Pelleted HIV-infected cells were resuspended in 10 ml of medium, transferred to a T25 cm^2 ^flask and incubated at 37°C in 5% CO_2_. 1 ml of medium was collected for intracellular p24 staining 5, 6, 7 and 8 days (where possible) post-infection. Pelleted cells (400 g for 5 min.) were resuspended in 100 μl IntraPrep™ Permeabilization Reagent Solution 1 (Beckman Coulter) for 15 min. at RT. (Room Temperature) to fix cells. Pelleted cells (200 g for 5 min.) were washed in 4 ml of PBS and resuspended in 100 μl IntraPrep™ Permeabilization Reagent Solution 2 to permeabilize cells during a 5 min. incubation at RT. Fixed and permeabilized cells were incubated with 5 μl of an anti-p24 PE-labelled monoclonal antibody (Beckman Coulter), or PE-labelled isotype control antibody (Beckman Coulter), for 15 min. at RT. Cells were washed with PBS to remove unbound antibodies, fixed for 30 min. at 4°C with 500 μl of fixing solution (PBS + 2% paraformaldehyde) before FACS analysis of intracellular p24 levels to determine the percentage of cells infected with HIV.

### Pfu-based PCR amplification and gel electrophoresis

Multiple cassette PCR amplicons were made with a Pfu-based method specificially developed for highly structured templates like multiple shRNA expression cassettes [46]. The primers were positioned 38 bp upstream and 21 bp downstream (inclusive) of the terminal cassettes/infinitely expandable cloning points, with the following sequences: forward (5'-3'): AGT TCT GCA CTC GGC CTC TG, and reverse (5'-3'): CCA TGG TCT GCA GTC GCT AG. The optimized Pfu-based PCR screening method consisted of the primers (20 pmol each), 1× Pfu Ultra II HS buffer (Stratagene), 3.5 mM MgCl_2 _(total), 10 mM dNTPs (each), ~10 ng of template (in as small a volume as possible), 2.5 μl DMSO (5%), 0.5 μl Pfu Ultra II HS (Stratagene), and H_2_O to a final volume of 50 μl. Each PCR was cycled at 1×: 95°C for 2 min., 35×: 95°C for 20 sec. | 66°C for 20 sec. | 72°C for 0.5 - 4 min. (depending upon template length), and 1× 72°C for 3 min. Samples were electrophoresed on 1% TAE agarose gels plus 0.01% SyberSafe stain (Invitrogen) at 200 V (limiting) for ~60 min. using a 150 × 245 mm tray, 3 mm wells with a Bio-Rad sub-cell model 192 apparatus. The Generuler 100 bp & 1 kb DNA ladders (Fermentas) were run as size markers along with a blended 1, 2, 3, 4, 5, and 6 shRNA cassette marker previously prepared by PCR amplification of the original plasmid preparations.

### Dot blotting

The presence of each shRNA encoding region within the PCR amplified multiple cassette samples was evaluated by Dot-blot using the same preparation as assayed by gel electrophoresis. 1 μl of each PCR sample was blotted on a positively charged nylon membrane (Ambion) by vacuum aspiration in the Bio-Dot^® ^SF Micro-filtration Apparatus according to the manufacturer's instructions (BioRad Laboratories). The membrane was UV auto cross-linked (using a Stratagene cross-linker) and hybridized with 50 ng of one of 6 unique 3' biotin-labelled, PAGE purified, DNA/LNA ('**L**ocked' **n**ucleic **a**cid) probes (Proligo) matched to each shRNA. Hybridization was in UltraHyb™ Oligo Hybridization buffer (Ambion) at 47°C overnight. The samples were detected using BrightStar™ BioDetect™ (Ambion) according to the manufacturer's instructions. All DNA/LNA probe sequences were (5' - 3'; + denotes the preceding nt. as an LNA base): **#3**: GAG+ CAGA+ TGAT+ ACAG+ TATT+ AC, **#8**: GAG+ CAGA+ AGAC+ AGTG+ GCAA+ TC, **#9**: TTG+ GAGA+ AGTG+ AATT+ ATAT+ AAC, **#2**: GAG+ CCAC+ CCCA+ CAAG+ ATTT+ AC, **#7**: ATG+ GCAG+ GAAG+ AAGC+ GGAG+ ACC, **#6**: CAG+ ATGG+ CAGG+ TGAT+ GATT+ GTC. LNA bases were approximately evenly distributed and were included to increase the target specific binding efficiencies.

### Modeling HIV-1 infection in the presence of a variable # of shRNAs

Our stochastic model tracked HIV infection in 343000 CD4+ T cells by quantifying the expansion or loss of transduced and untransduced cells over time and followed the development of mutations against each shRNA target site (**Manuscript in preparation**). Single mutations occurring in each shRNA target region either (**i**) had no impact on shRNA efficacy, (**ii**) decreased efficacy by 50%, or (**iii**) or conferred complete resistance, depending upon the location of the mutation within the the target site (the more central locations were considered more important). More than one mutation (anywhere) in a single shRNA target site also conferred complete resistance. Up to 3 recombination events per infectious cycle were also modeled to allow for further viral evolution beyond random mutations. Data points in each simulation were collected ~every 12 hours, up to ~13 years. Each cell could be infected by any of its 6 neighbours and lived ~2 days. Each cell that died from infection was replaced by a new cell exiting from the thymus, or through cell division of a neighbouring cell. The probabilities of the two replacement rates reflected the greater likelihood of CD4+ T cell expansion and the considerable involution of the thymus in adults and processes of peripheral homeostasis in adults [58]. Infected cells died at the same rate as production of new cells to maintain constant cell numbers. A proportion of all cells were selected to be long-lived to represent latency and maintain a constant source of virus. 20% of cells ejected from the thymus contained the integrated multiple shRNAs at proportions governed by the specified conditions in each scenario. The positions of deleted shRNAs and all other interactions were governed by chance with an underlying probability. Simulations were run using Matlab v.7 (The MathWorks Inc, Natick MA, USA).

## Competing interests

This work was done by employees of Johnson and Johnson Research (JJR), except for J. M. M. who performed consulting for JJR.

## Authors' contributions

GJM and TLA conceived the experiments. AJA and SWS performed the HIV-1 challenge assays. GJM, YY, AT, ABJ, AJA, MLM, MPB, FAE, and SWS prepared the clonal populations. AT performed the PCR assays and YY performed the dot blot assays. JMM, TLA and GJM performed the modeling. GJM and TLA analyzed and interpreted the results and wrote the manuscript.
